# Phase II Open Label Study of Valproic Acid in Spinal Muscular Atrophy

**DOI:** 10.1371/journal.pone.0005268

**Published:** 2009-05-14

**Authors:** Kathryn J. Swoboda, Charles B. Scott, Sandra P. Reyna, Thomas W. Prior, Bernard LaSalle, Susan L. Sorenson, Janine Wood, Gyula Acsadi, Thomas O. Crawford, John T. Kissel, Kristin J. Krosschell, Guy D'Anjou, Mark B. Bromberg, Mary K. Schroth, Gary M. Chan, Bakri Elsheikh, Louise R. Simard

**Affiliations:** 1 Departments of Neurology, Pediatrics, Neonatology and General Clinical Research Center, University of Utah School of Medicine, Salt Lake City, Utah, United States of America; 2 CBS Squared, Inc, Fort Washington, Pennsylvania, United States of America; 3 Departments of Molecular Pathology and Neurology, Ohio State University Medical Center, Columbus, Ohio, United States of America; 4 Departments of Neurology and Pediatrics, Wayne State University School of Medicine, Detroit, Michigan, United States of America; 5 Departments of Neurology and Pediatrics, Johns Hopkins University School of Medicine, Baltimore, Maryland, United States of America; 6 Department of Physical Therapy and Human Movement Sciences, Feinberg School of Medicine, Northwestern University, Chicago, Illinois, United States of America; 7 Division of Pediatric Neurology, Hôpital Sainte-Justine Montréal, Montréal, Québec, Canada; 8 Department of Pediatrics, University of Wisconsin School of Medicine, Madison, Wisconsin, United States of America; 9 Department of Biochemistry and Medical Genetics, University of Manitoba, Winnipeg, Manitoba, Canada; Columbia University, United States of America

## Abstract

Preliminary in vitro and in vivo studies with valproic acid (VPA) in cell lines and patients with spinal muscular atrophy (SMA) demonstrate increased expression of *SMN*, supporting the possibility of therapeutic benefit. We performed an open label trial of VPA in 42 subjects with SMA to assess safety and explore potential outcome measures to help guide design of future controlled clinical trials. Subjects included 2 SMA type I ages 2–3 years, 29 SMA type II ages 2–14 years and 11 type III ages 2–31 years, recruited from a natural history study. VPA was well-tolerated and without evident hepatotoxicity. Carnitine depletion was frequent and temporally associated with increased weakness in two subjects. Exploratory outcome measures included assessment of gross motor function via the modified Hammersmith Functional Motor Scale (MHFMS), electrophysiologic measures of innervation including maximum ulnar compound muscle action potential (CMAP) amplitudes and motor unit number estimation (MUNE), body composition and bone density via dual-energy X-ray absorptiometry (DEXA), and quantitative blood SMN mRNA levels. Clear decline in motor function occurred in several subjects in association with weight gain; mean fat mass increased without a corresponding increase in lean mass. We observed an increased mean score on the MHFMS scale in 27 subjects with SMA type II (p≤0.001); however, significant improvement was almost entirely restricted to participants <5 years of age. Full length SMN levels were unchanged and Δ7SMN levels were significantly reduced for 2 of 3 treatment visits. In contrast, bone mineral density (p≤0.0036) and maximum ulnar CMAP scores (p≤0.0001) increased significantly.

**Conclusions:**

While VPA appears safe and well-tolerated in this initial pilot trial, these data suggest that weight gain and carnitine depletion are likely to be significant confounding factors in clinical trials. This study highlights potential strengths and limitations of various candidate outcome measures and underscores the need for additional controlled clinical trials with VPA targeting more restricted cohorts of subjects.

**Trial Registration:**

ClinicalTrials.gov

## Introduction

Spinal muscular atrophy (SMA) is the most common inherited motor neuron disease, and a leading cause of infant and childhood mortality [1], [2]. With an incidence of 1 in 10,000 births, it is an autosomal recessive disorder associated with severe neuromuscular weakness and premature death in the majority of patients [3]–[6]. More than 96% of affected individuals demonstrate a homozygous deletion/mutation involving exon 7 in *SMN1* (survival motor neuron 1), resulting in the biochemical deficiency of the SMN protein, part of a complex that functions in the assembly of small nuclear ribonucleoprotein particles (snRNP) [7], [8]. A genomic duplication at this locus has resulted in a nearly identical gene, *SMN2* (survival motor neuron 2). *SMN2* differs from *SMN1* by a nucleotide substitution that promotes exon 7 exclusion. Consequently, SMN2 produces a fraction of the identical full length protein. Phenotypic variation in SMA correlates with the number of *SMN2* gene copies and the level of SMN protein in cells [9]–[15]. The broad range of phenotypes has led to classification into clinical types including the most common severe infantile form (SMA type I), non-ambulatory variants of intermediate severity (SMA type II), and ambulatory variants (SMA types III, IV). The majority of subjects develop symptoms in infancy or early childhood.

An opportunity for therapeutic intervention has arisen from the discovery of small molecule compounds which target *SMN2* gene copies present in all SMA patients to produce increased amounts of full-length SMN protein. Several compounds demonstrated to up-regulate SMN expression in SMA patient-derived cell lines, including valproic acid (VPA), sodium phenylbutyrate (NaPB) and hydroxyurea (HU), have been in clinical use for decades. These histone deacetylase (HDAC) inhibitors variably increase expression of many genes, including the SMN gene [16]–[20]. Preliminary in vivo data in human subjects supports up-regulation of SMN by both VPA and NaPB [21], [22]. VPA up-regulates *SMN2* expression at the promoter via inhibition of HDAC2, and both VPA and HU appear to alter splicing to increase full-length SMN protein [20], [23]. VPA has demonstrated neuroprotective properties on glutamate-induced excitotoxicity via up-regulation of alpha-synuclein and increases neurite outgrowth in vitro[24], [25]. Both VPA and NaPB have been reported to increase survival in ALS animal models [26], [27]. More recently, VPA administration in an SMA mouse model resulted in apparent improved motor function, larger evoked motor potentials, less degeneration of spinal motor neurons and improved neuromuscular junction innervation in treated animals compared to age-matched controls [28], [29]. Finally, two small open label trials of VPA in human subjects have reported modest strength or functional benefit in a subset of those patients [30], [31]. Such observations support the potential benefit of small molecule therapeutics not only for SMA, but for other forms of motor neuron disease, such as ALS. This preliminary in vivo and in vitro evidence encouraged us to proceed with an open label study of VPA in SMA patients as a first step towards more formal efficacy studies. Our primary objectives were to determine the safety of VPA in SMA patients and to assess the utility of a number of exploratory outcome measures for future clinical trials.

## Methods

The protocol for this trial and supporting CONSORT checklist are available as supporting information; see Checklist S1 and Protocol S1.

### Study population

Subjects participating in a natural history study at the University of Utah were recruited for an open label study of VPA (Clinicaltrials.gov ID NCT00374075). All subjects at least 2 years of age receiving <16 hours/day ventilator support were invited to participate. Fifty-eight subjects were enrolled in the natural history study. At the start of VPA study enrollment, thirteen type I subjects were deceased, required full-time mechanical ventilation, or were less than 2 years of age. Two subjects were excluded for prior noncompliance with natural history study visits. Two subjects declined due to the burden of study visits. Three additional subjects declined due to perceived risks. Enrollment was then opened in the order of calls received to recruit 4 additional subjects. Type I subjects were not specifically excluded but enrollment was discontinued following recruitment of 40 type II and type III subjects. Two subjects had a severe phenotype (SMA type I ages 2–3 years), 29 subjects had an intermediate phenotype (SMA type II ages 2–14 years) and 11 had a mild phenotype (SMA type III ages 2–31 years). Additional details of baseline study population characteristics are shown in Tables S1 and S2. The progress of all participants through the trial is diagrammed in Figure S1.

### Consent and adverse event grading

Written informed consent (subjects ≥18 years), parental consent (subjects <18 years) and assent (subjects ≥7 years) were obtained for all subjects. The study was approved by the University of Utah Institutional Review Board and General Clinical Research Center Advisory Committees. Adverse events were graded using Common Terminology Criteria for Adverse Events v3.0 (CTCAE v3.0). An independent Data and Safety Monitoring Committee provided oversight for the study.

### Study design

Inclusion criteria were age ≥2 years and confirmed genetic diagnosis of SMA. Exclusion criteria were ventilator support for ≥16 hours or concomitant medications with known hepatotoxicity or potential benefit in SMA. Each subject had to complete ≥2 natural history assessments within a 3–6 month period to qualify for enrollment in the VPA trial. Treatment assessments were performed at 3 (V1), 6 (V2) and 12 (V3) months. Divalproex sodium coated particles (Depakote® sprinkle capsules, 125 mg per capsule) were administered in divided doses two to three times daily sufficient to maintain overnight trough levels of 50–100 mg/dL. Dosing was typical of that used in epilepsy patients (15–50 mg/kg/day). Laboratory testing, which included a basic chemistry profile, CBC with platelets, transaminases, carnitine profile, amylase, lipase, and trough VPA levels, was performed at baseline, 2–3 weeks following initiation, and at each treatment visit.

Primary outcome measures were laboratory safety and adverse event data. Exploratory outcome measures included change from baseline assessments of motor function, pulmonary function (subjects ≥5 years), degree of denervation via maximum ulnar compound motor action potential (CMAP) and motor unit number estimation (MUNE) values, dual-energy X-ray absorptiometry (DEXA) of body composition and bone density and quantitative assessment of SMN mRNA.

Gross motor function was assessed with the Modified Hammersmith Functional Motor Scale for SMA (MHFMS). This scale contains the same 20 gross motor items as the original clinical scale, but was modified for use in the research setting [32], [33]. A version of this scale has been successfully used in a multicenter trial with SMA subjects [34]. Each item is scored from 0–2, with a hierarchy of skills ranging from rolling to sitting to standing. Unfortunately, this scale as it stands in its present form is inadequate for testing subjects across the full range of SMA phenotypes, due to significant floor and ceiling effects for subjects with SMA types I and III, respectively (additional details available at http://www.smaoutcomes.org). The degree of denervation in the hand was estimated using maximum ulnar CMAP amplitude and MUNE negative peak area values [12], [35]. In children ≥5 years, pulmonary function testing (PFT) was performed in an accredited pediatric laboratory, and included forced vital capacity (FVC), forced expiratory volume in 1 second (FEV1), and maximum expiratory and inspiratory pressures (MEP and MIP, respectively). We analyzed absolute values rather than corrected values due to concerns regarding accurate measurements of length due to contractures in many subjects. Norland DEXA XR-36 software version 3.3.1 for small subjects was used to assess whole body composition and bone mineral density and content.

SMN2 copy numbers were determined as previously described [11]. Whole blood was drawn into PAXgene tubes at baseline and each visit. Quantitative SMN mRNA levels were performed with slight modifications to the prior protocol [36]. Assays were carried out on a 7500 Real Time PCR system (Applied Biosystems). To increase efficiency on this apparatus, full-length SMN was amplified by primers 5′-GCTTTGGGAAGTATGTTAATTTCATGGT-3′ (exon 6) and 5′-TGTGAGCACCTTCCTTCTTTTTGAT-3′ (exon 7), and detected by the 5′FAM-TCTGAAACCCATATAATAGCC-MGBNFQ-3′ exon 6–7 junction probe. Delta-7 (Δ7) SMN was detected as published [36]. Human RPLPO (large ribosomal protein) and PGK1 (phosphoglycerate kinase 1) were run as endogenous controls. Patients missing baseline data or who had only one treatment visit were excluded from this analysis. Results are reported as relative amounts of full-length (fl) or Δ7SMN transcripts normalized against the relative amount of RPLPO.

### Statistical Analysis

The analysis of variance (ANOVA) test was used to evaluate the differences between means for function tests. When a factor level is empty and only two levels exists, ANOVA defaults to t-test. Linear regression analysis was used to examine the relationship between function parameters and genetic variables at baseline. The dependent variables in the linear regressions were evaluated for normality using the Shapiro-Wilk test and the paired t-test was used to test change from baseline in follow-up. If the dependent variable was not normally distributed then an appropriate transformation was used. A two-sided p-value less than 0.05 was considered statistically significant.

## Results

All 42 subjects completed *at least* 6 months of treatment. Five subjects discontinued treatment after the V2 visit at 6 months due to drug-related side effects, as detailed below. The remaining 37 subjects continued on treatment for a full year.

### Evaluation of safety and tolerability

For the most part, VPA was well tolerated and drug-related side effects mild. Grade I mood alteration was reported in three subjects. Grade II weight gain associated with functional decline led to discontinuation of VPA after V2 (6 months) in 2 subjects (both 9 year old girls, one with type II, the other with type IIIa but non-ambulatory). Poor compliance or lack of perceived benefit led to discontinuation in 3 additional subjects after V2 (2 siblings with type II, a 5 year old girl and 7 year old boy, and a poorly compliant 3 year old boy with type II who repeatedly refused oral dosing). One subject developed grade IV acidosis and carnitine depletion with respiratory failure requiring intubation and mechanical ventilation (3 year old type I subject). No deaths occurred during the study. Mean overnight (minimum 12 hour) trough VPA levels were 52 mg/dL (V1), 64 mg/dL (V2), and 59 mg/dL (V3). Abnormal laboratory values at baseline and during treatment are depicted in table 1; grade I elevations in transaminases without clinical correlate were present at baseline in 3 subjects and in 20 subjects (48%) for at least one visit. Transient grade I thrombocytopenia occurred in 2 subjects in association with illness. Grade I anemia was observed in 8 subjects at baseline and 19 subjects (45%) on treatment. Grade I leukopenia was frequent (36%) but absolute neutrophil counts remained >1.0×10^3^/uL in all subjects. Two subjects had low carnitine levels at baseline and a substantial proportion of the first thirteen subjects enrolled demonstrated reductions in total or free plasma carnitine within the first 3 months of treatment (Figure S2). In two subjects, this was associated with transient worsening of gross motor function which reversed with carnitine supplementation. Our Data Safety and Monitoring Board recommended supplementation of carnitine 50 mg/kg/day in all subjects subsequent to the first interim safety analysis.

**Table 1:** Percent of Subjects With Abnormal Lab Values Before and During Treatment All Patients (n = 42).

Laboratory Parameter	Baseline Incidence	Treatment Incidence
ALT	7%	19%
AST	10%	38%
WBC	12%	36%
HGB	17%	45%
HCT	17%	48%
Platelets	0%	5%
Neutrophils	36%	57%

ALT = alanine aminotransferase; AST = aspartate aminotransferase; WBC = total white blood count; HGB = hemoglobin; HCT = hematocrit.

### Evaluation of gross motor function

Gross motor function was assessed using the MHFMS in a subgroup of 27 of 29 non-ambulatory SMA II subjects; one 14.9 year old subject with severe contractures who was no longer able to sit unsupported, and one uncooperative 2.8 year-old toddler were not evaluated with the MHFMS. Mean age of the remaining 27 subjects was 4.58 years. All subjects completed V1 and V2 visits and 25 (86%) completed all three visits. V1 was performed at 3.2 (2.3–4.3) months; V2 at 6.8 (5.5–9.9) months and V3 at 13 (8.7–15.6) months following treatment initiation. Mean MHFMS scores improved by 2.15; 2.92; and 4.65 at V1, V2 and V3 visits respectively, compared to baseline (table 2, p≤0.001). Motor function in type II subjects was then defined as stable, deteriorated or improved using a three or six point change in the MHFMS score. Each time point was examined for frequency of that change from baseline for the entire cohort (table 3, part I). We further explored the effect of age on change in motor function (table 3, part II). Of note, 8/16 (50%) children <5 years of age achieved at least a 6 point improvement after 1 year, while no children ≥5 years of age demonstrated a six point improvement. The most significant change in scores was observed in 5 SMA type II children ages 25–34 months at enrollment who gained from 8–15 points. Table 4 further documents the effect of age on change in motor function for those children who achieved at least a 3 point improvement on the MHFMS.

**Table 2:** Change in MHFMS Score by Time Point.

Time	Type II Subjects				Type III Subjects			
	Avg Change	Range	SD	p-value^1^	Avg Change	Range	SD	p-value^1^
V1	2.15 N = 27	−5/7	3.02	0.0010	1.0 n = 10	−2/9	3.20	NS
V2	2.92 N = 26	−4/13	3.78	0.0006	1.4 n = 10	−4/11	3.92	NS
V3	4.65 N = 23	−6/15	4.76	0.0001	1.44 n = 9	−2/9	3.24	NS
Baseline	13.7 N = 27	0/35	8.22		34.4 n = 10	10/40	9.40	

1 P-Values based upon paired t-test.

V1 = 3 months; V2 = 6 months; V3 = 12 months; SD = standard deviation, NS = not significant.

**Table 3:** Deterioration vs. Improvement in MHFMS score, SMA type II.

Part I. Three vs. Six point change, All type II Subjects				
Criteria	Change	V1	V2	V3
Three-Point		N = 27	N = 26	N = 23
	Deterioration	2 (7%)	1 (4%)	1 (4%)
	Stable	14 (52%)	12 (46%)	6 (26%)
	Improvement	11 (41%)	13 (50%)	16 (70%)
Six-Point				
	Deterioration	0	0	1 (4%)
	Stable	23 (85%)	21 (81%)	14 (61%)
	Improvement	4 (15%)	5 (19%)	8 (35%)

Part II. Deterioration vs. Improvement by Age, Six-Point Change				
Criteria	Change	V1	V2	V3
Under 5 years		N = 18	N = 17	N = 16
	Deterioration	0	0	0
	Stable	14 (78%)	12 (71%)	8 (50%)
	Improvement	4 (22%)	5 (29%)	8 (50%)
5+ years		N = 9	N = 9	N = 7
	Deterioration	0	0	1 (14%)
	Stable	9 (100%)	9 (100%)	6 (86%)
	Improvement	0	0	0

**Table 4:** Deterioration vs. Improvement by Age: Three-Point Change, SMA type II.

Criteria	Change	V1	V2	V3
Under 5 years		N = 18	N = 17	N = 16
	Deterioration	1 (6%)	0	0
	Stable	9 (50%)	6 (35%)	2 (13%)
	Improvement	8 (44%)	11 (65%)	14 (87%)
5+ years		N = 9	N = 9	N = 7
	Deterioration	1 (11%)	1 (11%)	1 (14%)
	Stable	5 (56%)	6 (67%)	4 (57%)
	Improvement	3 (33%)	2 (22%)	2 (29%)

Gross motor function was assessed using the MHFMS in a subgroup 10 of 11 ambulatory SMA III subjects. Increase in MHFMS scores in this cohort was limited by ceiling effects since several scored maximally at baseline (table 2).

### Evaluation of pulmonary function measures

Pulmonary function testing was performed in 14 subjects ≥5 years of age, comprised of 10 type II subjects and 4 type III subjects. We observed a significant improvement in MIP values at the V1 and V2 visits, and in FVC, FEV1 and MIP values at the V3 visit in type II subjects (table 5).

**Table 5:** Change in Pulmonary Function Tests from Baseline by Time Point.

Time	Type II Subjects N = 10			Type III Subjects N = 4	
	Avg Change Range	SD	p-value^1^	Avg Change Range	SD
**V1 (3 months)**					
FVC (L)	0.04	0.10	NS	0.10	0.27
	−0.06/0.22			−0.10/0.41	
FEV1 (L)	0.00	0.12	NS	−0.03	0.09
	−0.13/0.22			−0.12/0.05	
MIP (cm H_2_O)	−11.0	8.9	p = 0.0059	−5.7	20.0
	−24.0/1.0		−26/14.0		
MEP (cm H_2_O)	18.0	27.9	NS	−9.3	23.2
	−7.0/81.0			−35.0/10.0	
**V2 (6 months)**					
FVC (L)	0.07	0.12	NS	0.09	0.23
	−0.12/0.21			−0.11/0.40	
FEV1 (L)	0.04	0.15	NS	−0.03	0.14
	−0.27/0.22			−0.10/0.22	
MIP (cm H_2_O)	−16.6	17.4	p = 0.0212	−13.0	17.0
	−39.0/20.0			−38.0/0.0	
MEP (cm H_2_O)	16.9	24.5	NS	7.25	17.9
	−4.0/74.0			−7.0/30.0	
FVC (L)	0.12	0.14	p = 0.0472	0.28	0.20
	−0.12/0.26			−0.14/0.42	
FEV1 (L)	0.12	0.14	p = 0.0467	0.26	0.21
	−0.12/0.26			−0.12/0.41	
MIP (cm H_2_O)	−16.6	17.7	p = 0.0481	−16.6	30.2
	−35.0/18.0			−38.0/4.7	
MEP (cm H_2_O)	14.3	16.9	NS	1.3	23.6
	−3.0/47.0			−15.0/18.0	

FVC = forced vital capacity; FEV1 = forced expiratory volume in 1 second; MIP = maximal inspiratory pressure; MEP = maximum expiratory pressure.

### Evaluation of body composition and bone density

Total lean body mass was stable but fat mass increased 38% between the baseline and the one year visit in type II subjects; this is in contrast to a 14% increase in type III subjects (table 6). We observed progressive increases in total body bone mineral density and content (BMD, BMC) across all treatment visits in type II subjects (p≤0.0034) and at V2 and V3 in type III subjects (p≤0.0066). When change in MHFMS score was analyzed by change in fat mass, those with three or six point gains demonstrated a lower gain in fat mass than those who were stable or deteriorated (p = NS; table 7).

**Table 6:** Change in Lean and Fat Mass and Bone Density by Time Point.

Time	Type II Subjects N = 29			Type III Subjects N = 10		
	Avg Change^1^	SD	p-value^2^	Avg Change^1^	SD	p-value^2^
**V1 (3 months)**						
Lean mass, grams	123	973	NS	−267	1865	NS
Fat mass, grams	1094	1034	0.0003	1269	1844	0.078
Total BMD, g/cm^2^	0.05	0.06	0.0003	0.02	0.05	NS
Total BMC, grams	33.6	54.4	0.0036	71.0	80.0	0.0287
**V2 (6 months)**						
Lean mass, grams	−366	1174	NS	184	1582	NS
Fat mass, grams	2579	2013	<0.0001	2101	2213	0.0149
Total BMD, g/cm^2^	0.05	0.06	<0.0001	0.03	0.03	0.0066
Total BMC, grams	99.5	68.1	<0.0001	90.5	76.2	0.0045
**V3 (12 months)**						
Lean mass, grams	−212	1029	NS	322	960	NS
Fat mass, grams	3680	2470	<0.0001	2596	3193	0.0406
Total BMD g/cm^2^	0.07	0.06	<0.0001	0.07	0.04	0.0011
Total BMC, grams	145	66.4	<0.0001	124.2	98.1	0.0052

1 Range at baseline in Table S1.

2 P-values based upon paired t-test.

BMD = bone mineral density; BMC = bone mineral content; g/cm^2^ = grams/centimeter squared; SD = standard deviation, NS = not significant.

**Table 7:** Change in MHFMS Score by Change in Fat Mass.

Criteria	Change	V1	V2	V3
Three-Point				
	Deterioration	2320	7541	9502
		N = 2	N = 1	N = 1
	Stable	1173	1995	4459
		N = 14	N = 12	N = 6
	Improvement	825	2318	2918
		N = 11	N = 13	N = 16
P-value^1^		0.73	0.22	0.09
Six-Point				
	Deterioration			9502.0
		N = 0	N = 0	N = 1
	Stable	1287	2584	3624
		N = 23	N = 21	N = 1
	Improvement	189	1460	2839
		N = 4	N = 5	N = 8
P-value^1^		0.10	0.27	0.18

1 P-value based upon ANOVA.

### Evaluation of electrophysiologic measures of denervation

CMAP amplitude increased over baseline at each visit. The increase in mean values was significant for V2 and V3 in type II subjects and for all visits for type III subjects (table 8, p≤0.0127). There was also a significant difference in CMAP values by change in MHMFS. At V1, subjects showing at least a three point improvement demonstrated higher CMAP values, indicating the best response in those with less severe denervation (table 9; p = 0.03 ). Changes in MUNE negative peak area or amplitude values were not significant.

**Table 8:** Change in Maximum Ulnar CMAP Amplitude and MUNE Negative Peak Area from Baseline by Time Point.

Time	Type II Subjects (n = 29)				Type III Subjects (n = 10)			
	Avg Change	Range	SD	p-value^1^	Avg Change	Range	SD	p-value^1^
V1 (3 months)								
CMAP (mV)	0.12	−0.98/1.26	0.38	0.0909	1.03	−0.10/2.50	0.95	0.0074
MUNE (µVms)	−3.03	−95.0/10.0	18.6	NS	−0.50	−38.0/53.0	28.2	NS
V2 (6 months)								
CMAP (mV)	0.23	−0.96/1.12	0.39	0.0041	1.20	0.23/3.56	1.13	0.0085
MUNE (µVms)	−5.59	−117.0/7.0	22.2	NS	−16.4	−93.0/17.0	33.8	NS
V3 (12 months)								
CMAP (mV)	0.35	−0.52/1.58	0.54	0.0031	1.44	−0.24/3.75	1.35	0.0127
MUNE (µVms)	−8.58	−126.0/10.0	26.3	NS	−17.7	−93.0/17.0	51.0	NS

1 P-values based upon paired t-test.

SD = standard deviation, NS = not significant.

**Table 9:** Average Maximum Ulnar CMAP vs. Three-point and Six-point Change in MHFMS Score.

Criteria	Change	CMAP (mV)	CMAP (mV)	CMAP (mV)
		V1	V2	V3
3-Point				
	Deterioration	0.46	0.47	0.55
		N = 2	N = 1	N = 1
	Stable	1.59	1.70	1.22
		N = 14	N = 12	N = 6
	Improvement	2.05	2.11	2.00
		N = 11	N = 13	N = 16
P-value^1^		0.03	0.07	0.29
6-Point				
	Deterioration			0.55
		N = 0	N = 0	N = 1
	Stable	1.52	1.68	1.33
		N = 23	N = 21	N = 14
	Improvement	2.67	2.58	2.61
		N = 4	N = 5	N = 8
P-value^1^		0.29	0.24	0.24

1 P-value based upon ANOVA.

Maximum ulnar CMAP (compound muscle action potential amplitude).

### Evaluation of SMN2 copy number and quantitative measures of SMN mRNA in whole blood samples

A linear regression analysis was performed on the transformed MHFMS scores (square root transformation) to evaluate the influence of genetic variables, including SMN2 copy number and SMN mRNA levels on MHFMS (Table 10). The results indicate no statistically significant relationship between MHFMS score and SMN mRNA levels, but do indicate a positive relationship between SMN2 copy number and SMA type with MHFMS score. Subjects with 3 SMN2 copies had an average of 0.67 flSMN compared to 0.79 flSMN in subjects with 4 SMN2 copies (Table S3). Δ7SMN levels were on average 1.04 and 1.15 for SMN2 copies 3 and 4, respectively. These results indicate no relationship between SMN mRNA levels and SMN2 copy number. There was a trend toward an increased relative amount of Δ7SMN transcripts per SMA type (0.91±0.47 for type 2 vs. 1.42±1.11 for type 3; p = 0.053). The mean relative amount of flSMN and Δ7SMN transcripts was 0.67±0.29 and 0.91±0.47 for type II and 0.77±13 and 1.42±1.11 for type III, respectively. Baseline measures point to the high degree of inter-patient variability in the relative amount of SMN transcripts, as previously reported [36], [37].

**Table 10:** Linear Regression of Square Root of MHFMS Total Score Versus Genetic Variables.

Term	Estimate	Std Error	t Ratio	Prob>|t|
Intercept	−0.77	1.07	−0.71	0.4818
flSMN	−0.19	0.82	−0.23	0.8177
Δ7SMN	−0.68	0.45	−1.50	0.1455
SMN2	0.52	0.25	2.05	0.0497
Type	2.35	0.48	4.93	<.0001

There were no significant changes in the relative amount of flSMN transcripts in response to drug treatment. There was a significant decrease in Δ7SMN transcripts between baseline and V2 (p = 0.0053) and V3 (p = 0.0429) for type II subjects (table 11). Similar results were observed when using PGK1 as endogenous control to normalize for amount of input cDNA template and RT-PCR efficiency (baseline data in Table S3, treatment data not shown). Finally, there was no relationship between decreased Δ7SMN transcripts and any physiological or functional measure.

**Table 11:** Change in Quantitative SMN mRNA levels by Time Point.

Time	Type II Subjects (n = 25)				Type III Subjects (n = 11)			
	Avg Change	Range	SD	p-value^1^	Avg Change	Range	SD	p-value^1^
**V1**	N = 25				N = 11			
flSMN	0.10	−0.19/2.00	0.42	NS	−0.02	−0.17/0.04	0.06	NS
Δ7SMN	0.42	−1.17/10.91	2.26	NS	−0.47	−2.68/0.65	0.98	NS
**V2**	N = 25				N = 11			
flSMN	−0.01	−0.38/0.52	0.17	NS	−0.04	−0.25/0.12	0.11	NS
Δ7SMN	−0.35	−1.82/0.60	0.57	0.0053	−0.46	−3.17/1.25	1.26	NS
**V3**	N = 19				N = 9			
flSMN	−0.01	−0.34/0.33	0.16	NS	−0.30	−0.14/0.10	0.06	NS
Δ7SMN	−0.32	−1.61/1.01	0.64	0.0429	−0.30	−3.25/1.63	1.34	NS
Baseline values								
flSMN	0.67	0.05/1.27	0.29		0.77	0.63/1.05	0.13	
Δ7SMN	0.91	0.05/2.14	0.47		1.42	0.41/4.29	1.11	

1 P-value based upon ANOVA.

## Discussion

In this open label study, we enrolled a relatively heterogeneous group of SMA subjects, although we focused recruitment predominantly on children with SMA types II and III. Our experience emphasizes the need to stratify subjects by SMA type, age and current gross motor abilities in future trials since the majority of outcome measures examined in this study were optimal for only a subset of subjects which was highly dependent on the subject's age, motor disability and the overall degree of medical fragility.

This study provides evidence that VPA can be used safely in SMA subjects >2 years of age, as long as carnitine status is closely monitored. VPA is known to alter enteral absorption, inhibit biosynthesis and secondarily deplete carnitine from muscle by direct binding and excretion in the urine [38]. Given the diminished lean body mass in SMA subjects, an increased susceptibility to carnitine depletion is not surprising. Decreased nutritional intake and depleted whole body carnitine stores related to an associated abnormality in fatty acid metabolism may be contributing factors [39], [40]. Thus, treatment with VPA in the absence of close monitoring and supplemental carnitine therapy could increase risks for further muscle weakness or fatal hepatotoxicity [41]. In this study, we did not observe any serious clinical or laboratory evidence of hepatotoxicity with careful monitoring and supplementation but grade I elevations of transaminases were common. Infants and children less than two years of age, a group not included in the current study, theoretically have a greater risk of such complications.

The apparent positive effect of treatment on bone density is surprising since prolonged VPA treatment has been associated with decreased bone density in other populations [42]–[45]. Whether the observed increased bone density is spurious or relates to increased weight gain, weight bearing or other factors is unclear. The significant increase in fat mass in the absence of an increase in lean mass is concerning given the detrimental impact weight gain could have on gross motor function and potential long-term consequences for general health. Weight gain was not uniform across the population; rather, non-ambulatory subjects ≥5 years, or those already with such a tendency appear to be at greatest risk. Notably, children demonstrating the most significant improvement in gross motor function gained less fat mass compared to those who displayed stable or deteriorated motor function.

Maximum CMAP amplitudes showed a statistically significant increase from baseline, while MUNE values did not change. This would imply that axonal reinnervation did not account for the change, but that the observed changes may be due to reinnervation of muscle via “sprouting” from remaining motor units or a direct trophic effect on muscle. If future controlled trials confirm that increased CMAP amplitude is a consistent marker of biologic response that correlates with improvement in motor function, then this outcome measure could prove a powerful surrogate biomarker for future clinical trials.

Changes in pulmonary function were observed; however, the significance is limited by the small number of subjects ≥5 years in each cohort. Nonetheless, PFT measures are important with regard to clinical outcomes in patients with SMA, so other means of reliably determining pulmonary function in younger children is an important goal.

We had hypothesized that VPA might increase flSMN and Δ7SMN mRNA levels in blood samples in light of prior data demonstrating increased mRNA and protein levels in patient-derived cells [16]–[20]. When analyzed as a group, we observed a significant decrease in the relative amount of Δ7SMN mRNA in SMA type II subjects while flSMN transcripts were unchanged. However, flSMN and Δ7SMN mRNA levels fluctuated throughout drug treatment, with patients showing increased, decreased or unaltered relative amounts of both transcripts. This variability contrasts with the fairly stable SMN mRNA levels observed in untreated patients [21], [36], [37]. Consistent with previously published data, these results are suggestive of a VPA response and SMN mRNA could help discriminate between non-responders and responders [21]. Furthermore, the implication of decreased relative Δ7SMN mRNA levels in the absence of changes in flSMN would be to increase the ratio of flSMN to SMNΔ7, the major discriminator between SMA subjects and unaffected controls/carriers when using relative measures of SMN mRNA, suggesting that these changes could be important. Finally, in order for SMN mRNA levels in blood to inform us about VPA effects in spinal cord, there must be a significant relationship between SMN mRNA and a clinical outcome measure. We did not observe any relationship between changes in Δ7SMN and any of the outcome measures in this pilot open label study. One reason for this may be that blood may not be the appropriate tissue to screen for biomarkers of SMA. Alternatively, extensive variability in SMN mRNA levels during treatment trials may mask significant relationships in a sub-set of SMA patients. Fluctuations in SMN mRNA levels may be amplified by fluctuations in transcript levels produced by endogenous controls used normalize for the amount of input template and efficacy of RT-PCR. VPA is a non-specific drug target and may itself affect expression of genes used as endogenous controls. We observed similar results with two endogenous controls (RPLPO and PGK1) and do not have any evidence to suggest that these genes are affected by VPA treatment. Nonetheless, there is a need for an absolute quantification method to measure SMN mRNA in order to remove variability introduced by the use of endogenous controls to normalize data.

In conclusion, this study provides good evidence that VPA can be used safely in SMA subjects over 2 years of age in the setting of close monitoring of carnitine status. This being said, further studies of VPA in infants and young children are needed to better assess safety in this more vulnerable cohort. This study provides evidence in support of improvement in gross motor function in younger non-ambulatory type II children. This finding was unexpected and it is unclear whether this improvement reflects a therapeutic drug effect, maturation or increased cooperation on improved scores in the youngest subjects. These data suggest that further studies with VPA are warranted, although clearly such findings must be replicated in randomized, controlled efficacy studies. The data presented here emphasizes the benefit of a trial design with a less heterogenous population of younger SMA subjects, particularly in light of significantly increased fat mass and lack of apparent benefit of VPA treatment in older non-ambulatory subjects. Furthermore, our experience indicates that the MHFMS scale alone is not adequate to measure motor function in ambulatory SMA subjects, prompting us to explore other measures to assess functional change in this population. Future clinical trials targeted to this cohort should generate additional informative data as to the usefulness of PFTs, timed test modules, and biomarker data including electrophysiologic measures of denervation, which appear to have promise in this cohort.

## Supporting Information

Protocol S1 Clinical trial protocol summary
(0.11 MB DOC)

Figure S1 CONSORT Flowchart
(4.21 MB TIF)

Figure S2 Free carnitine levels at enrollment and subsequent follow-up for the first thirteen subjects
(0.31 MB TIF)

Checklist S1 CONSORT Checklist
(0.06 MB DOC)

Table S1 (0.08 MB DOC)

Table S2 (0.05 MB DOC)

Table S3 (0.04 MB DOC)
