# Supplementary material for: Phase II Open Label Study of Valproic Acid in Spinal Muscular Atrophy
Source: PLoS One. 2009 May 14;4(5):e5268. doi: 10.1371/journal.pone.0005268 (PMC2680034; doi:10.1371/journal.pone.0005268)
Supplement: Protocol S1 — Clinical trial protocol summary (0.11 MB DOC) [file pone.0005268.s001.doc]

**Open Label Phase II Trial of Valproic Acid and Carnitine**

**in Patients with Spinal Muscular Atrophy**

**PRINICIPAL INVESTIGATOR:** Kathryn J. Swoboda, M.D.

University of Utah

Department of Neurology

30 North 1900 East #3R210

Salt Lake City, Utah 84132

phone (801) 585-1676

fax (801) 587-9346

beeper (801) 914-6771

email [swoboda@genetics.utah.edu](mailto:swoboda@genetics.utah.edu)

**PROJECT SPONSOR:** Families of SMA

**CLINICAL TRIAL MANAGER:** Sandra P. Reyna, M.D.

University of Utah

Department of Neurology

30 North 1900 East #3R210

Salt Lake City, Utah 84132

phone (801) 585-1676

fax (801) 587-9346

beeper (801) 329-7599

email [sreyna@genetics.utah.edu](mailto:sreyna@genetics.utah.edu)

**BIOSTATISTICIAN:** Charles B. Scott, Ph.D. Director, Children’s Hospital of

Philadelphia

Biostatistics and Data Management

3535 Market Street 10^th^ Floor

Suite 1032 and 1035

Philadelphia, PA 19104

**DATA MANAGEMENT:** Bernie LaSalle

General Clinical Research Center

University of Utah

30 North 1900 East #4R210

Salt Lake City, UT 84132-2411

**SPONSOR OF IND:** Kathryn J. Swoboda, M.D.

**Background and Introduction**

Spinal muscular atrophy (SMA) is an autosomal recessive motor neuron disease characterized by diffuse weakness and muscular atrophy. It is one of the most common neuromuscular conditions, and a leading cause of hereditary infant mortality. Patients most frequently present in infancy and early childhood, but the clinical spectrum of onset and severity is broad, ranging from the infant with congenital joint contractures and ventilator dependence to adult onset of proximal muscle weakness. This clinical heterogeneity increases the inherent challenges in clinical trial design for the evaluation of promising new therapies. Our work and that of others have demonstrated that SMA is a progressive motor neuron disease, as reflected by loss of function as well as maximum compound motor action potential amplitudes and motor unit number estimates in a distally innervated hand muscle over time. Disease progression can occur rapidly in SMA 1 infants prior to reaching a stable albeit significantly weak endpoint, while patients with SMA types 2 and 3 often demonstrate a more slowly progressive course, with stable function preserved over many years.

Homozygous deletion of the survival motor neuron 1 (SMN1) gene is found in more than 94% of SMA cases regardless of subtype, and plays a primary role in disease pathogenesis. The SMN1 gene resides in a duplicated inverted region on chromosome 5q. A near identical copy of the SMN1 gene, designated SMN2, contains a single nucleotide change which alters splicing, and drastically reduces the efficiency by which exon 7 is incorporated, leading to a less than full length mRNA and protein product. However, SMN2 transcription produces a small percentage of full length SMN protein. An increased number of SMN2 gene copies, with the resulting increase in full-length mRNA levels and protein expression, is associated with a less severe disease phenotype in humans, and phenotypic rescue in an SMN knockout mouse model.

The SMN protein appears to play an important role in spliceosomal small nuclear ribonucleoproteins (snRNP) biogenesis, although the precise means by which a deficiency of the SMN protein directly affects neuronal viability remains unclear. The identification of the unique mechanism by which phenotype is modified in SMA, via SMN protein expression from a near identical gene copy, has led to efforts to identify specific compounds which could shift splicing in the SMN2 message toward a higher rate of exon 7 inclusion or up-regulate SMN protein expression via an increase in SMN2 gene expression. We have recently developed an SMN protein assay in patient monocytes and platelets that appears to correlate with SMA severity, and is stable in repeated assays over up to a one year period of time.

At this time, there are no registered pharmacological treatments for SMA. However, several compounds have recently been identified that appear to up-regulate SMN gene expression or alter gene splicing to produce an increased ratio of full length to exon-7-deleted SMN mRNA. Several of these compounds are readily available for use in patients, and have a proven safety record in the treatment of other disorders. The purpose of this proposal is to assess the safety and tolerability of one such compound, valproic acid (VPA), and assess its effects on SMN protein and mRNA levels *in vivo*.

Valproic acid (2-propylpentanoic acid, dipropylacetic acid, Depakene®, Depakote®, Depakote®ER, Abbott Pharmaceuticals) is a carboxylic acid with demonstrated efficacy in the treatment of primarily generalized, complex partial, and absence seizures. Although initially marketed in the treatment of epilepsy, subsequent clinical studies have demonstrated its efficacy for treatment of migraine and bipolar disorder. It comes in multiple dosage forms, including liquid, sprinkle capsules and scored tablets. Dosage initiation begins at 10 mg/kg/day, then is typically increased by a rate of 5 mg/kg/week up to a maximum of 60 mg/kg/day. Plasma levels are monitored to achieve a target range of 50 to 100 micrograms/mL. Thousands of patients with epilepsy have been treated with valproic acid (VPA) over the past decade, and it has a well-established safety profile. Treatment is discontinued in approximately 2 to 6% of patients for side effects which include asthenia, headache, nausea and other gastrointestinal symptoms, weight gain, tremor, somnolence, dizziness, and hair loss. Rare life-threatening and fatal reactions have been reported which include hepatotoxicity (with increased risk reported for children less than age two years, and in those with congenital metabolic disorders or on multiple anticonvulsant medication), and pancreatitis. Hepatic dysfunction is an absolute contraindication to treatment with VPA.

At the VIIth International Families of Spinal Muscular Atrophy Meeting in June 2003, two laboratories reported preliminary data supporting the potential benefit of VPA on SMN protein and mRNA levels. Drs. L. Brichta and B. Wirth presented data demonstrating that the level of full-length *SMN2* mRNA/ protein increased 2-4-fold in fibroblast cell lines derived from SMA patients treated with therapeutic doses (0.5-500 *µ*M) of VPA. In addition, VPA increased SMN protein levels through transcription activation in organotypic hippocampal brain slices from rats. Drs. C. Sumner and K. Fischbeck reported that VPA significantly increases exon 7-containing SMN mRNA transcript and protein levels in three SMA Type 1 fibroblast cell lines after 24 hours of treatment. This increase was accentuated after 3 days of treatment. VPA treatment also increased the number of gems, the intra-nuclear structures containing SMN protein. They deomonstrated that VPA promoted correct splicing of the *SMN2* gene and activated the *SMN* promoter, indicating that the increase in full-length SMN levels in response to VPA may be due both to enhancement of correct splicing and to an increase of SMN2 transcription*.* Results from these studies seem to indicate that VPA and related compounds deserve further investigation as potential treatment for SMA.

VPA is a histone deacetylase inhibitor, and its effect on SMN gene expression has been hypothesized to be associated with this function. VPA has been reported to induce fetal hemoglobin synthesis in children with sickle cell anemia, and is purported to play a role in upregulation of gene expression of other genes in vivo. Thus, clinical interest in the use of this compound for novel treatment approaches is increasing.

**Study Objectives:**

1. To assess safety and tolerability of VPA in SMA patients greater than 2 years of age
2. To look for a potential in vivo effect of VPA on SMN mRNA and protein levels in patient white blood cells, monocytes and platelets at routinely used clinical doses
3. To assess effect on potential clinical outcome measures, including assessment of gross motor function, strength, fat and lean body mass and bone density, and electrophysiologic measures of denervation, including ulnar CMAP and MUNE studies.

**Patient Selection Criteria:**

Patients must have a diagnosis of SMA, confirmed by genetic testing. Only patients with confirmed homozygous deletion of the SMN1 gene are eligible for enrollment. We propose to recruit patients already enrolled in our natural history study: Clinical and Genetic Analysis of Spinal Muscular Atrophy 8751-01, for the VPA trial. Only patients 2 years of age and older will be eligible for the VPA trial. Additional exclusion criteria include patients taking any medications with known hepatotoxicity, congenital metabolic disorders or on multiple anticonvulsant medications. Eligible patients are recruited predominantly via referral from the national support organization Families of SMA. We anticipate the no new patients will have to be recruited as we already have 60 patients enrolled in the natural history study. Not all of these patients will be eligible due to age restraints. We will selectively choose those patients who have most reliably followed up with repeat evaluations in the natural history study. Information regarding our new VPA study will be posted on the families of SMA web site so that the SMA community will be aware of the trial. Once IRB approval occurs, a press release will be posted by Families of SMA and will provide information regarding the study.

**Design:**

This is an open label phase 1 clinical trial to assess safety, tolerability and potential effect on SMN mRNA and protein in vivo of a compound in which preliminary evidence supports a potential effect on SMN levels in vitro.

Phase 1: Screening Phase

Forty patients with SMA types II and III will be recruited into the study following appropriate written informed consent. Entry criteria includes confirmation of homozygous deletion in the SMN1 gene, and clinical assessment. Only SMA patients older than the age of 2 years will be included in the VPA trial. Patients with known liver disease are excluded from participation. Patients currently enrolled in other treatment trials, or on medications which may interact with VPA are also excluded. Although we won’t specifically exclude SMA type I subjects, recruitment is targeted to enrollment of a total of forty subjects with SMA type II and III.

Phase 2: Run-in Phase

Patients will have been assessed on three separate occasions to obtain baseline information regarding severity of their diagnosis at time 0, 3 months (10 to 14 weeks post 1^st^ assessment) and 6 months (10 to 14 weeks post 2^nd^ assessment) via their involvement in the natural history study. Baseline parameters prior to inclusion in the study group will be obtained on their third visit, and include the following: CBC with platelets, AST, ALT, amylase, lipase and carnitine panel. If carnitine levels are low, carnitine supplementation will be instituted at 50 mg/kg/day and levels repeated prior to enrollment in the study. Patients with baseline liver transaminases more than 1.5 x normal are automatically excluded from the trial.

Phase 3: Treatment Phase

Treatment with VPA will be instituted at 10 mg/kg/day divided two to three times daily using routinely available dosage forms, and increased at 5 mg/kg/week to reach a standard dosage range of 20 to 30 mg/kg/day. Readily available dosage forms include 125 spinkle capsules;125, 250 and 500 mg tablets, and syrup in a dose of 250 mg/5 ml. Plasma levels will be assessed at 2 to 4 weeks and 3 months post institution of treatment, and dosing adjusted as tolerated so that levels fall within the standard anticonvulsant range of 50 to 100 milligrams/mL.

**Study Procedure Outline**

Clinical assessment at each time point includes general examination, vital signs, growth parameters including height, weight and head circumference. In addition, they will undergo assessment of exploratory clinical outcome measures included in the natural history protocol (for which all patients are dually consented), including whole body DEXA for lean/fat mass and bone density, maximum ulnar CMAP and MUNE studies, motor function as assessed using a modified version of the Hammersmith functional motor scale for SMA, and PFTs including spirometry, MIP and MEP in all subjects > 5 yrs of age.

Visit 1(baseline): Clinical assessment, CBC with platelets, AST, ALT, amylase, lipase, carnitine levels, SMN protein and mRNA levels, maximum ulnar CMAP and MUNE studies, whole body DEXA for lean/fat mass and bone density, and measures of gross motor function appropriate to the function of that patient.

Blood draw for safety assessment (2 to 4 weeks post institution of therapy): CBC with platelets, AST, ALT, amylase, lipase, valproic acid, carnitine levels – these levels may be obtained via their local hospital laboratory, and do not require a clinical assessment

Visit 2 (3 months): Clinical Assessment, CBC with platelets, AST, ALT, amylase, lipase, valproic acid, carnitine levels, SMN protein and mRNA levels, maximum ulnar CMAP and MUNE studies, whole body DEXA for lean/fat mass and bone density, and measures of gross motor function appropriate to the function of that patient.

Visit 3 (6 months): Clinical Assessment, CBC with platelets, AST, ALT, amylase, lipase, valproic acid, carnitine levels, SMN protein and mRNA levels, maximum ulnar CMAP and MUNE studies, whole body DEXA for lean/fat mass and bone density, and measures of gross motor function appropriate to the function of that patient.

Visit 4 (12 months): Clinical Assessment, CBC with platelets, AST, ALT, amylase, lipase, valproic acid, carnitine levels, SMN protein and mRNA levels, maximum ulnar CMAP and MUNE studies, whole body DEXA for lean/fat mass and bone density, and measures of gross motor function appropriate to the function of that patient.

Clinical testing on the first day of the study visit will include the following: a general physical examination including tests of blood pressure, breathing rate and oxygen level, heart rate, and temperature, and growth parameters including height, weight, head circumference, chest circumference, and abdominal circumference. We estimate this will take about 20 minutes. Nerve conduction testing will be performed at each visit (to determine maximum ulnar CMAP and MUNE). This testing involves attaching electrodes (½ inch rectangular adhesive pads) over muscles in the hand. The electrodes taped over muscles in the hand will be connected to an EMG machine to record responses. Patients will lay in the position most comfortable for him/her. A nerve will be activated at the wrist and around the elbow by applying brief electrical shocks. The time required for the nerve conduction testing and monitoring is approximately 60 minutes.

Blood samples will be obtained either immediately before or after the nerve conduction testing. If the patient is going to be sedated, the blood draw will be done while the patient is still under sedation. Three to six milliliters of blood is needed at each visit to check for safety monitoring and drug levels. If patients elect to have their DNA collected for genetic studies, an additional five to ten milliliters will be collected during the course of the study. DEXA scan will also be performed on this day. The total time of testing on the first day, including all of the testing above, is approximately 90 minutes.

Prior to the patient’s arrival for each study visit they will be given a 3 day dietary/supplement record, which they will asked to complete and bring with them. This information will be reviewed with them by a dietician either on their arrival or on the first day of testing. If patients forget to bring their completed dietary record, they will be asked to complete it the following week and return it promptly.

Clinical testing on the second day includes the following: A physical therapist will perform a functional assessment using either the Modified Hammersmith Functional Motor Scale – SMA (cohort 1) or the Ambulatory Functional Motor Scale for SMA (cohort 2). Functional testing will take 15 to 30 minutes. Patients 5 years of age or older will receive pulmonary function testing. A technician will perform the pulmonary function testing, which includes spirometry, Maximum Inspiratory Pressure (MIP), and Maximum Expiratory Pressure (MEP). This testing will take approximately 30 minutes.

Participation in this study will include 4 visits and at least 6 blood tests over a 12 month period. Each visit will be two to three days long. The first day will be for travel if needed, the second day will include clinical exam, nerve conduction testing and collection of blood samples, and all functional testing will be performed on the third day. Follow-up Study Visits after beginning therapy will be at 3 months (Visit 1), 6 months (Visit 2) and 12 months (Visit 3) when the patient will stop drug. All evaluations will be performed at each follow-up visit.

*Outcome Measures*

1. Safety labs: CBC with platelets, AST, ALT, amylase, lipase, carnitine profile, basic metabolic panel, and VPA level (after baseline screening visit)
2. Quantitative assessment of SMN mRNA from blood samples
3. Functional Status/Strength as assessed via Modified Hammersmith Functional Motor Scale (MHFMS) only (type II) or with the Ambulatory SMA 3 functional motor scale (still under final development), which contains additional add-on modules including timed tests and additional gross motor items (type III ambulatory)
4. Pulmonary Function Testing: spirometry, Maximum Inspiratory Pressure, Maximum Expiratory Pressure (> 5 yrs)
5. Max CMAP amplitude/area: ulnar nerve/hypothenar eminence
6. Ulnar MUNE
7. Growth and vital sign parameters
8. Nutritional Status (DEXA, growth parameters and dietary analysis)
9. Bone Density (DEXA)

*Safety Laboratories:*

To ensure that patients are tolerating the study medication safely, blood chemistries and drug levels will be drawn at each study visit and closely monitored throughout the course of the study. Safety Laboraties will include: Complete Blood Count (CBC) with platelets, AS Transferase (AST), AL transferase (ALT), Amylase, Lipase, Sodium (NA^+^) levels, Potassium (K^+^) levels, Chloride (Cl^+^) levels, Glucose levels, Carbon Dioxide (CO2), Blood Urea Nitrogen (BUN) levels, Creatinine, VPA level and Carnitine profile. These laboratories require a minimum 5 mls of blood.

*Functional Assessment*

Pilot studies at the University of Utah and Montreal have validated excellent test-retest reliability and stability for the modified version of the published Hammersmith scale. This test is optimized for SMA type 2 patients and non-ambulatory type 3 patients (cohort 1). For SMA type 3 patients (cohort 2) we will use the Ambulatory SMA Functional Scale for standers/walkers.

Each child will be tested using the Modified Hammersmith Functional Motor Scale for Children with Spinal Muscular Atrophy (MHFMS-SMA) at each visit. The MHFMS-SMA testing will be carried by the clinical evaluator and each assessment will be videotaped. This assessment consists of a hierarchical series of simple functional gross motor tasks requiring increasing amounts of strength to perform. The tasks mimic those that are part of many routine physical therapy sessions and/or daily activities. There are 20 items to be tested, and testing can take from 15-30 minutes depending on the child’s level of function and cooperation. Items are scored as follows: 0-unable to complete, 1-partially completes task, 2- completes task. The test total score is equal to 40.

Each child able to participate (ie able to stand and walk) will be tested using the Ambulatory Motor Scale for SMA at each visit. The testing will be carried by the clinical evaluator and each assessment will be videotaped. This assessment consists of a hierarchical series of simple and higher level functional gross motor tasks requiring increasing amounts of strength to perform. In addition, there are several timed functional tasks that will look at how quickly the child can finish a task. The tasks mimic those that are part of many routine physical therapy sessions and daily activities. Testing can take from 45-60 minutes depending on the child’s level of function and cooperation.

*Pulmonary Functional Testing*

Children older than 5 years will undergo a standardized pulmonary function testing (PFTs) including routine spirometry, maximum inspiratory pressure (MIP), and maximum expiratory pressure (MEP).

Pulmonary function testing will include spirometry and respiratory muscle force testing. Spirometry will be measured using standard testing techniques by a pulmonary function test (PFT) technician in a pulmonary function laboratory at each center. Tests will include spirometry, maximal inspiratory pressure (MIP) and maximal expiratory pressure (MEP) measurements. Spirometry assesses ventilatory function and includes the measurement of forced vital capacity (FVC), the forced expiratory volume in the first second (FEV1), and forced mid-expiratory flow (FEF25-75%). MIP and MEP testing assesses the aggregate force that the respiratory muscles can generate against an occlusion at the mouth. MIP is an index of diaphragm strength and is measured near residual volume. MEP measures the strength of the abdominal and intercostal muscles and is measured at total lung capacity. There is no discomfort performing this test. If a subject is unable or unwilling to perform the test, the testing will be discontinued.

Equipment includes a diagnostic spirometer that meets and is maintained based on American Thoracic Society guidelines^42^. In addition, this equipment has the software and hardware adaptations including an electronic pressure transducer or manometer and recording device that allow for the measurement of respiratory muscle pressures. Methods are standardized and based on the Pulmonary Function Laboratory Management and Procedure Manual published by the American Thoracic Society and the ATS/ERS Statement on respiratory muscle testing^43^. References values will be reported from each center.

*SMN mRNA Studies*

Dr. Louise Simard at the University of Montreal has developed an assay to measure mRNA levels in patient samples via quantitative techniques using real time RTPCR. She has shown that mRNA levels in whole blood demonstrate good stability and reproducibility over time when obtained and analyzed using the appropriate protocol. This biological marker will be evaluated in an exploratory fashion to see if it correlates with other outcome measures. For SMN mRNA quatitification, approximately 2.5 ml of whole blood will be drawn into PAXgene™ blood RNA vacutainer tubes (PreAnalytiX^TM^, a Qiagen/BD Company) that contain 7.5 ml of a RNA stabilization solution. RNA remains stable in this solution for at least 5 days at room temperature. PAXgene™ tubes will be shipped on ice to the Simard laboratory; we have determined that shipment on ice increases RNA yield. Packages must be labeled “store at 4^o^C upon receipt”.

*Electrophysiological Assessments (CMAP and MUNE)*

Little information is available on the relationship between the number of remaining healthy lower motor neurons and the degree of clinical weakness in SMA; moreover, the severity and timing of motor neuron loss in SMA is poorly understood. Two important physiological tests have recently been employed to investigate this: Maximum CMAP amplitude/area of ulnar nerve/hypothenar eminence and ulnar Motor Unit Number Estimation (MUNE). To determine the Maximum CMAP amplitude/area, surface electrodes are placed over the muscle as in routine nerve conduction studies and the maximal CMAP is determined following electrical stimulation of the motor nerve. The stimulating electrode can be moved to a different site along the nerve and another all-or-none single SMUP can be obtained. This process can be repeated at multiple sites along the nerve, and approximately 8-12 SMUPs can be summed to obtain an average. The MUNE value is the Maximal CMAP amplitude or area divided by the average single motor unit amplitude or area. There are no current techniques to actually count the number of motor neurons innervating a muscle: MUNE represents an estimate. However, MUNE is the only methodology available to make these estimates.

Preliminary studies at the University Utah have shown that there is a correlation between SMA type and the CMAP and MUNE values.

MUNE and maximum ulnar CMAP amplitude: We use the multiple point stimulation (MPS) technique to estimate the number of motor units innervating hypothenar muscles. Pilot studies at the University of Utah have been performed using a proprietary software program on the Advantage® A200 machine (no longer available). Further studies will be performed on the Comperio® from Compumedics, from which we have obtained comparable results, and for which multicenter assessment is ongoing. To ensure maximum CMAP is selected, the G1 electrode is moved for a minimum of up to 3 placements to attempt to reach prior maximum CMAP amplitude. Individual single motor unit potentials (SMUPs) are identified by obtaining all-or-none responses to low intensity stimulation. Ten consecutive observations of an individual SMUP are obtained to decrease the probability of multiplex units, and we attempt to identify at least 10 unique SMUPs. Values are expressed for CMAP and SMUP by both negative peak area and negative peak amplitude.

Conscious sedation: Sedation may be necessary or helpful in reducing excessive anxiety in some children, and will be performed under the supervision of the study site investigator with the assistance of trained nurses to assist with the administration of medications and monitoring patient’s vital signs.  An anesthesiologist or emergency-attending physician will be immediately available should an unexpected complication ensue during the procedure.  Patients will be NPO for several hours prior to the procedure as per routine hospital sedation guidelines.  The goal is conscious sedation, to minimize anxiety and voluntary movement but not to deeply sedate the patient or even put them to sleep. Use of sedation will be carefully determined by age and ability to cooperate. Patients will be sedated using either oral valium, or intranasal or oral midazolam (versed). Valium dosing range is 0.5-0.8 mg/kg/dose with a maximum dose of 10 mg. Midazolam dosing range is 0.1 – 0.3 mg/kg/dose (intranasally) or 0.2 - 0.5 mg/kg/dose (orally); children may receive up to one dose every ten to twenty minutes during the procedures (maximum 2 doses).  Possible complications could include respiratory compromise, hypotension or an unexpected paradoxical response to the medication, resulting in agitation. Heart rate, pulse oximetry, and blood pressure will be measured prior to the start of the study, monitored continuously during the study, and for 30 min following its completion or until the patient is adequately awake and has completely returned to baseline respiratory status. Suction equipment and oxygen will be available at the bedside if needed.

*Growth and Vital Sign Parameters*

Growth and vital sign parameters will be collected at each visit and will be important correlates to the other outcome measures.

Weight (kg) will be determined in a standardized method using electronic scales with less than 1% error. All scales will have been calibrated to Weights and Measures regulation. Height (cm) measurements will use a stadiometer for children who are standers and a portable plastic length board for measuring the children who are sitters. Head, chest, and abdominal circumference measurements will use a flexible plastic metric tape measure. A blood pressure cuff will be placed on their upper arm or lower leg to obtain a blood pressure using an automated electronic sphygnomanometer. Heart rate and saturation will be obtained using pulse oximetry, placed on a toe or a finger. A temperature will also be obtained using a digital ear thermometer.

*Dietary/Supplement Record and Nutritional Analysis*

Prior to each scheduled visit, patients will receive a 3-day dietary record and nutritional supplement record, which they will complete and bring with them. This information will be analyzed by a registered dietician, and reviewed with them either on their arrival or on the first day of testing for each visit. Results will be correlated with growth parameters. If patients neglect to bring the completed record, they will be asked to complete it the following week and return it promptly.

Dietary/supplement surveys and counseling will help ensure the following:

1. identify supplement/medication use that might disqualify them from the study
2. allow ongoing monitoring to minimize grossly excessive vitamin use or protein, fat, sugar intake
3. help monitor and avoid major changes in supplement use during the course of the study

*Genetic studies*

While genetic confirmation of 5q SMA is necessary for inclusion in this study, that information will come from the routine clinical evaluation of patients prior to enrollment. New genetic information obtained in our study may include the SMN2 copy number, a known modifier of SMA phenotype, which will be performed in the CLIA certified laboratory of Dr. Tom Prior at Ohio State University. We will also request consent to establish a DNA and cell line resource on each patient, their parents, and other family members if applicable, so as to have this resource available to perform further genetic studies. Such studies may be critical in identifying features which predict “responders” from “non-responders”, and assist in the identification of genetic modifier loci with potentially important future therapeutic applications. However, patients and parents may refuse to participate in the development of a permanent DNA and cell line database without affecting their eligibility for enrollment in this study.

*Other Laboratory Studies*

SMN copy number: Confirmation of homozygous SMN1 deletion and assessment of SMN copy number will be performed in the CLIA-certified molecular diagnostic laboratory at Ohio State University, and reviewed by Dr. Thomas Prior. Dosage determination of the SMN2 copy number is performed be a competitive PCR method using exogenous *in vitro* synthesized DNA internal standard. This takes about 2.5 ml of blood. Investigators and families will remain blinded to this data until completion of the trial.

**Safety Monitoring:**

Patients will be made aware of all potential risks during the informed consent process. Safety assessments will consist of monitoring and recording all adverse events and serious adverse events; the regular monitoring of parameters including hematology, liver function tests, vital signs, and general physical condition. Blood draws will be obtained during each study visit and between some study visits to monitor safety levels.

The Medical Monitor (MM) will review all laboratory data within a week of each visit and on as needed basis to determine whether any subject suffers evidence of organ damage or an adverse event, and to make decisions regarding dosage adjustments. All lab results will be provided on laboratory reports along with normal limits for each value. The MM will note any abnormalities and sign the case report form before returning to the Clinical Trials Manager. In addition, the MM will receive monthly reports summarizing any adverse events.

*Assessment and Reporting of Adverse Events (AE)s*

An adverse event is any physical or clinical change experienced by the patient.  This includes the onset of new symptoms and the exacerbation of pre-existing conditions.  All AEs will be recorded in the patient’s study records and on the appropriate CRF. The severity of the AE will be assessed; actions/outcomes (e.g., hospitalization, discontinuation of therapy, etc.) will also be recorded for each AE. Patients with adverse events will be monitored with relevant clinical assessments and laboratory tests as determined by the Medical Monitor. All adverse events will be followed to satisfactory resolution or stabilization of the event(s).

Any actions taken and follow-up results will be recorded in the patient’s source documentation.  Follow-up laboratory results will be filed with the patient’s source documentation.

The following definitions will be used:

Mild: no antidote therapy or prolongation of hospitalization is required.

Moderate: required change in therapy, specific additional therapy or an increase in hospitalization by at least 1 day.

Severe: potentially life threatening, causing permanent damage or requiring intensive medical care.

The onsite investigator will also assess the relationship of any adverse event to study procedure, based on available information. The following will be reported as adverse events: recurrent vomiting or gastrointestinal distress beyond the normal reflux that occurs in most SMA patients, evidence of hepatitis or pancreatitis as documented by abnormal laboratory findings and typical clinical symptoms such as gastrointestinal distress, jaundice, recurrent vomiting; skin rash suggesting a drug reaction, bone marrow suppression as documented by thrombocytopenia, anemia, or leucopenia in a range that is clinically relevant: total WBC < 3.0; neutropenia < 1.0; platelets < 100,000; hematocrit < 30; transaminases, AST, ALT, lipase or amylase > 3 x normal value for age, and hospitalization for any reason. Reflux, constipation, and chronic respiratory symptoms, including pneumonia, can be part of the normal phenotype associated with SMA. In addition, failure to gain weight appropriately or even weight loss or loss of motor functions in the context of an interim illness are common. These events will not be considered serious adverse events unless hospitalization is required, but will be managed appropriately in the usual fashion.

*Serious Adverse Events (SAEs)*

A serious adverse event (SAE) is defined by the FDA as "an event that is fatal or life threatening, permanently disabling, requires initial or prolonged hospitalization, causes congenital anomaly or requires intervention to prevent permanent impairment or damage."

Because these children are often hospitalized for respiratory illnesses, the medical monitor will be notified immediately regarding such hospitalizations, and in conjunction with the site co-investigator will help guide their local physician regarding whether or not to discontinue the medication.

The Common Toxicity Criteria (CTC) Version 2.0 grades 3 and 4 will be used as a way to operationalize the definition of serious adverse event which would lead to discontinuation of study medication.  For example, this would create the following STOP criteria using the planned screening labs:  Hemoglobin <8 g/dL; platelet count <50,000/mm3, absolute neutrophil count <1000/mm3, serum sodium <130 mmol/L, serum potassium >6 mmol/L, serum bicarbonate less than or equal to 10 mEq/dL, creatinine > 3 times upper limit of normal (ULN); liver function tests, amylase or lipase > 5 times ULN). Any cause of death will of course be considered a serious adverse event.

All serious adverse events, whether or not deemed procedure-related or expected, will be reported by the investigator to the IRB and the Clinical Trials Manager within 24 hours (one working day) by telephone.  A written report will follow as soon as possible, which includes a full description of the event and any sequelae.  This includes serious events that occur any time after the inclusion of the patient in the study until completion of the last visit.  Notification to the FDA will occur within 24 hours of notification of the event.

Concomitant Interventions: The patient’s medical management will continue as usual. No prescription drugs will be prohibited if medically indicated. However, the patient will be prohibited from participating in other clinical trials while in this study. Nutritional monitoring via 3 day dietary surveys and counseling at each visit will emphasize standard dietary guidelines appropriate for each child’s age and weight, and attempt to minimize grossly excessive supplemental vitamin or protein intake or changes in nutritional interventions over the course of the study unless medically indicated. Herbal preparations will be discouraged since their contents may not be known. In the case of a serious adverse event, the Principal Investigator will make the decision as to whether or not to withdrawn the patient in consultation with the Data Safety and Monitoring Committee (DSMC).

**Data and Safety Monitoring Committee**

*Composition*

The DSMC will consist of 6 members who will be completely independent of all investigators and have no financial, scientific or other conflict of interest with the study. A quorum will be 4 voting members. The Principal Investigator and Data Management Biostatistician will be non-voting members.

Colin B. Van Orman, MD Medical Monitor/Pediatric Neurologist

Thomas. F. Pajak, Ph.D Biostatistician

Robert M. Taylor, MD Ethicist

Michael Workman, PT Pediatric Physical Therapist

Natalie Mellem, PT Pediatric Physical Therapist

Jacob V. Aranda, MD, PhD Clinical Neonatologist/Pharmacologist

*Confidentiality*

All materials, discussions and proceedings are completely confidential. The DSMC will receive data without any patient identifiers, only study numbers. The only link between patient names and study ID numbers will remain at the individual study site.

Genetic Research: All study patients enrolled in this study will be concurrently enrolled in the natural history study (#8751 Clinical and Genetic Analysis of Spinal Muscular Atrophy). While genetic confirmation of SMA is necessary for inclusion in this study, that information will come from the natural history study and no new genetic information will be obtained in this study. Therefore, there is no need to address genetic guidelines in this study.

**Statistical Methods, Data Analysis and Interpretation:**

The analysis of variance (ANOVA) test will be used to evaluate the differences between means for function tests. Linear regression analysis will be used to examine the relationship between function parameters and genetic variables at baseline. The dependent variables in the linear regressions will be evaluated for normality using the Shapiro-Wilk test, and t-tests will be used to test change from baseline in follow-up. Analysis across follow-up will be performed using mixed effect analysis. A two-sided p-value less than 0.05 will be considered statistically significant. Biomarker data analysis: comparison of baseline SMN protein and mRNA levels (mean value as established from 3 pre-enrollment timepoints via baseline data established via their enrollment in the natural history study) will be made with each time point.

**Administrative Responsibilities**

Adam Craner, nurse practitioner, has the responsibility of enrolling new study patients. Data collection and management is performed by Adam Craner, the principal investigator Kathryn Swoboda and her clinical coordinator Mark Wride, in conjunction with Bernie LaSalle of the data management center. Patient samples and other patient information are given an identifying number and the code key is kept in a password protected computer. Patient files are stored according to number and are kept in locked files by the investigators.

**References** **and Appendices**
