# Supplementary figures and images for: Phase II Open Label Study of Valproic Acid in Spinal Muscular Atrophy
Source: PLoS One. 2009 May 14;4(5):e5268. doi: 10.1371/journal.pone.0005268 (PMC2680034; doi:10.1371/journal.pone.0005268)

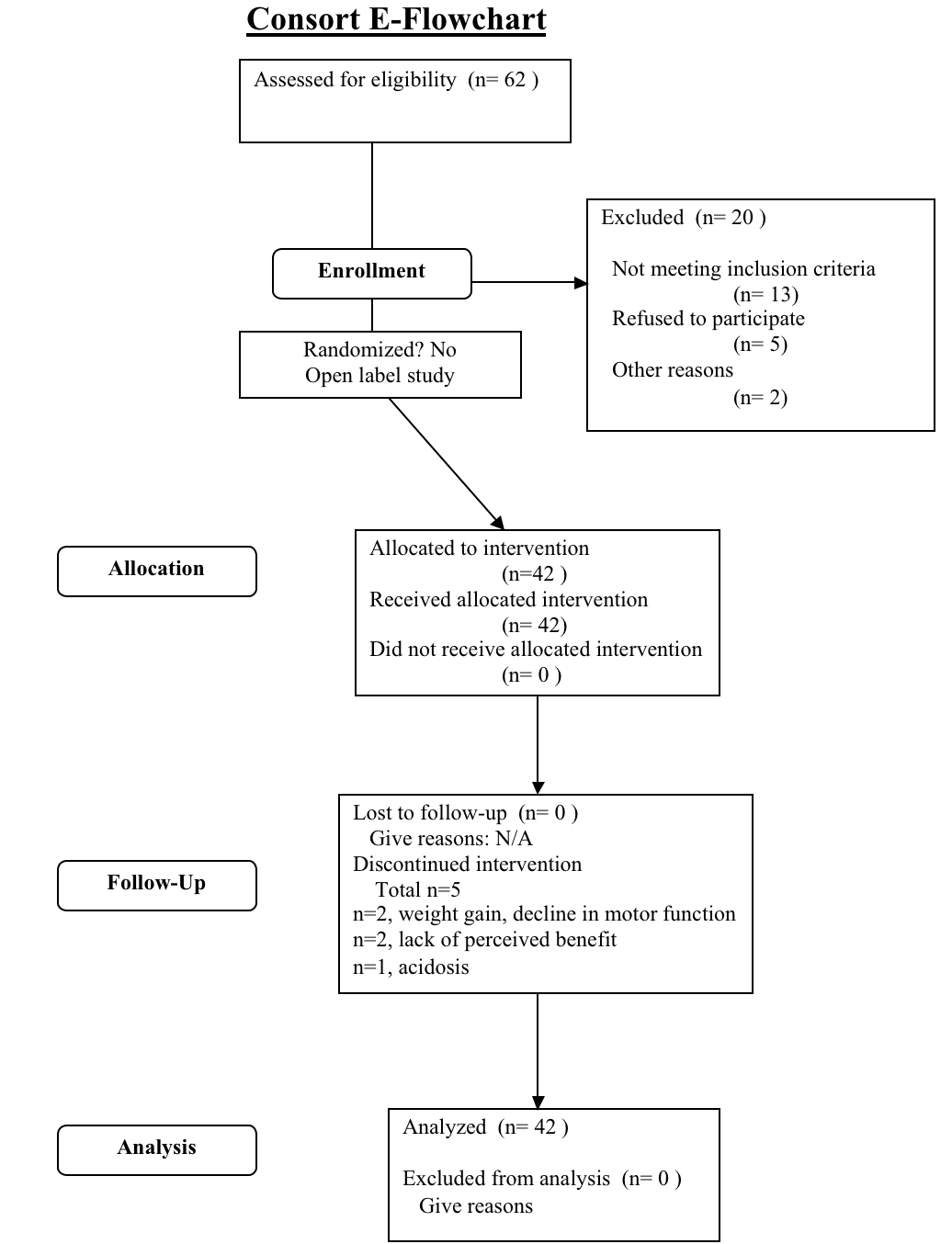

Supplement: Figure S1 — CONSORT Flowchart (4.21 MB TIF) [file pone.0005268.s002.tif]

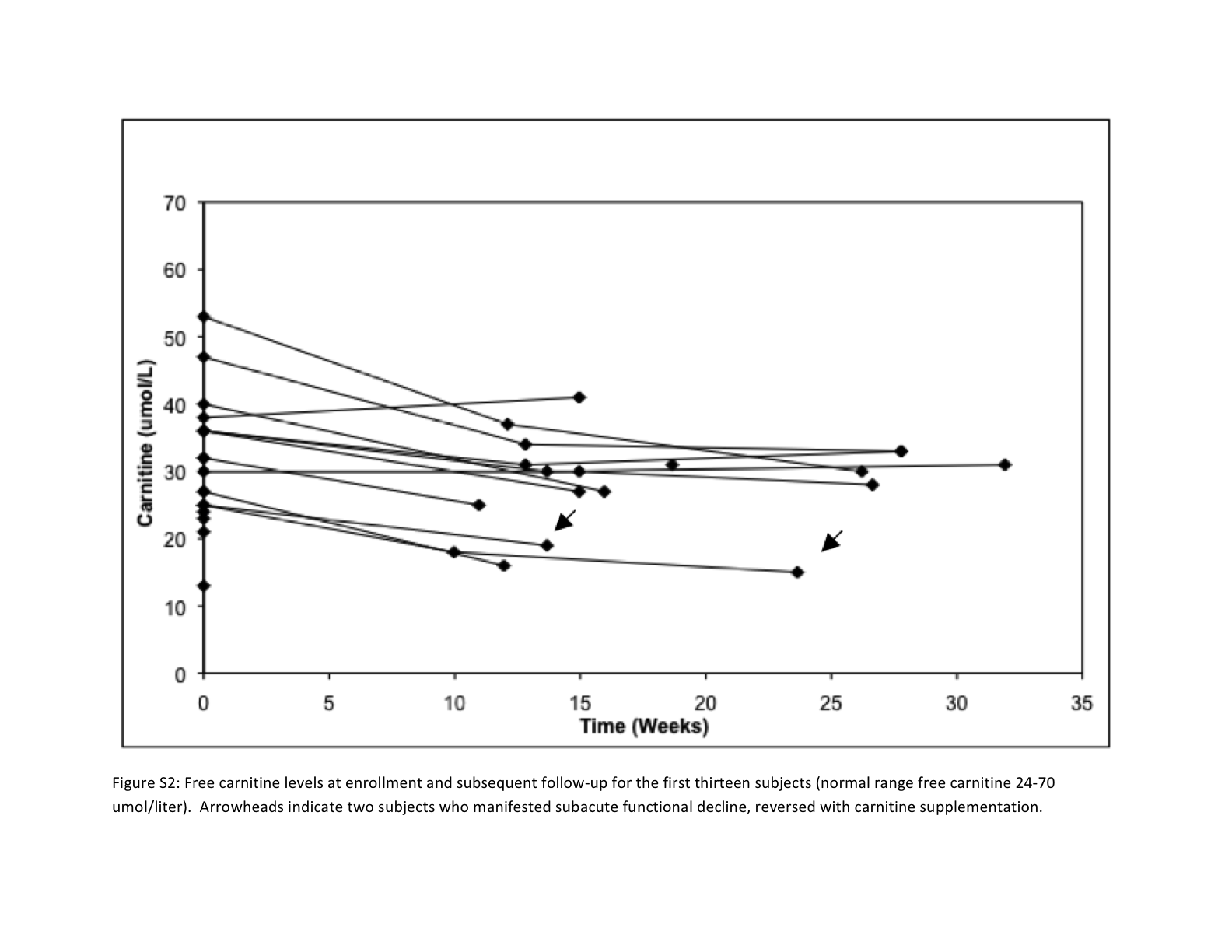

Supplement: Figure S2 — Free carnitine levels at enrollment and subsequent follow-up for the first thirteen subjects (0.31 MB TIF) [file pone.0005268.s003.tif]
