# Supplementary material for: Phase II Open Label Study of Valproic Acid in Spinal Muscular Atrophy
Source: PLoS One. 2009 May 14;4(5):e5268. doi: 10.1371/journal.pone.0005268 (PMC2680034; doi:10.1371/journal.pone.0005268)
Supplement: Table S1 — (0.08 MB DOC) [file pone.0005268.s005.doc]

| **Table S1: Baseline Characteristics by SMA type** | | | | |
| --- | --- | --- | --- | --- |
| SMA Type | Type 1 | Type 2 | Type 3 | Total |
| Number of subjects | N=2 | N=29 | N=11 | N=42 |
| Age (years) | | | | |
| Mean | 2.6 | 4.9 | 8.3 | 5.7 |
| Range | 2.1-3.2 | 2.0-14.9 | 2.6-31.0 | 2.0-31.0 |
| Weight (kg) | | | | |
| Mean | 12.6 | 16.8 | 21.7 | 17.8 |
| Range | 12.6 | 9.2-58.0 | 11.6-61.8 | 9.2-61.8 |
| SMN2 Copies | | | | |
| 2 | 1 (50%) |  |  | 1 ( 2%) |
| 3 | 1 (50%) | 22 (76%) | 8 (73%) | 31 (74%) |
| 4 |  | 7 (24%) | 2 (18%) | 9 (22%) |
| 5 |  |  | 1 ( 9%) | 1 ( 2%) |
| Total Bone Mineral Density (BMD, g/cm^2^) | | | | |
| N | 2 | 29 | 10 | 41 |
| Mean | 0.70 | 0.85 | 0.85 | 0.84 |
| SD | 0.12 | 0.11 | 0.08 | 0.11 |
| Range | 0.61-0.78 | 0.68-1.09 | 0.74-0.99 | 0.61-1.09 |
| Lumbar Spine levels L2-L4 Bone Mineral Density (L2-L4 BMD, g/cm^2^) | | | | |
| N | 2 | 27 | 10 | 39 |
| Mean | 0.27 | 0.32 | 0.56 | 0.84 |
| SD | 0.02 | 0.07 | 0.26 | 0.18 |
| Range | 0.26-0.29 | 0.20-46 | 0.31-1.11 | 0.20-1.11 |
| Total Body Bone Mineral Content (BMC, g) | | | | |
| N | 2 | 29 | 10 | 41 |
| Mean | 418.8 | 605.7 | 1120.4 | 722.2 |
| SD | 4.10 | 271.5 | 803.7 | 501.0 |
| Range | 415.9-421.7 | 283-1713 | 496.6-2851.0 | 283.0-2851.0 |
| Lumbar Spine Levels L2-L4 Bone Mineral Content (BMC, g) | | | | |
| N | 2 | 27 | 10 | 39 |
| Mean | 5.5 | 6.3 | 15.3 | 8.6 |
| SD | 1.0 | 2.1 | 14.2 | 8.2 |
| Range | 4.8-6.2 | 3.0-10.5 | 5.3-52.9 | 3.0-52.9 |
| Total Body Lean Mass (g) | | | | |
| N | 2 | 29 | 10 | 41 |
| Mean | 4160 | 5914.5 | 8284.7 | 6407 |
| SD | 947 | 1867.4 | 4565.1 | 2909 |
| Range | 3490-4830 | 3504-11156 | 4327-18147 | 3490-18147 |
| Total Body Fat Mass (g) | | | | |
| N | 2 | 29 | 10 | 41 |
| Mean | 7447.5 | 9907.9 | 15130.2 | 11061.6 |
| SD | 672.5 | 7631.0 | 12564.7 | 8999.4 |
| Range | 6972-7923 | 3431-42601 | 4901-43047 | 3431-43047 |
| Full-length SMN (flSMN) referenced to po (flSMN/po) | | | | |
| N | 0 | 27 | 11 | 38 |
| Mean |  | 0.67 | 0.77 | 0.70 |
| SD |  | 0.29 | 0.13 | 0.25 |
| Range |  | 0.05-1.27 | 0.63-1.05 | 0.05-1.27 |
| Delta 7 SMN (Δ7SMN) referenced to po (Δ7SMN/po) | | | | |
| N | 0 | 27 | 11 | 38 |
| Mean |  | 0.91 | 1.42 | 1.06 |
| SD |  | 0.47 | 1.11 | 0.74 |
| Range |  | 0.05-2.14 | 0.41-4.29 | 0.05-4.29 |

flSMN=full length survival motor neuron mRNA levels; Δ7SMN=delta 7 SMN (missing exon 7) survival motor neuron mRNA levels; po = human RPLPO, large ribosomal protein, run as an endogenous control.

Table S1 shows baseline characteristics of the study population with regard to SMA type, age, baseline weight, SMN2 copy number, fat mass and lean mass, total body and lumbar spine (L2-L4 levels ) bone mineral density (BMD) and bone mineral content (BMC), full length survival motor neuron mRNA levels (flSMN) and delta 7 SMN (Δ7SMN) survival motor neuron mRNA levels absent exon 7.
