# Supplementary material for: Phase II Open Label Study of Valproic Acid in Spinal Muscular Atrophy
Source: PLoS One. 2009 May 14;4(5):e5268. doi: 10.1371/journal.pone.0005268 (PMC2680034; doi:10.1371/journal.pone.0005268)
Supplement: Table S2 — (0.05 MB DOC) [file pone.0005268.s006.doc]

| Table S2. Baseline Characteristics by SMA Type | | | | |
| --- | --- | --- | --- | --- |
| SMA Type | Type I | Type 2 | Type 3 | Total |
| Maximum Ulnar Compound Muscle Action Potential Amplitude (CMAP, mV) | | | | |
| N | 2 | 29 | 10 | 41 |
| Mean | 0.25 | 1.52 | 6.80 | 2.74 |
| SD | 0.01 | 1.26 | 2.16 | 2.77 |
| Range | 0.24-0.26 | 0.29-5.86 | 3.01-9.70 | 0.24-9.70 |
| Modified Hammersmith Functional Motor Scale for SMA Score (MHFMS score, 0-40) | | | | |
| N | 0 | 27 | 10 | 37 |
| Mean |  | 13.7 | 34.4 | 19.3 |
| SD |  | 8.2 | 9.4 | 12.6 |
| Range |  | 0-35 | 10-40 | 0-40 |
| Forced Vital Capacity (FVC, L) | | | | |
| N | 0 | 10 | 4 | 14 |
| Mean |  | 0.86 | 2.52 | 1.34 |
| SD |  | 0.30 | 0.86 | 0.82 |
| Range |  | 0.41-1.24 | 1.76-3.56 | 0.41-3.56 |
| Forced Expiratory Volume in One Second (FEV1, L) | | | | |
| N |  | 10 | 4 | 14 |
| Mean |  | 0.78 | 2.24 | 1.19 |
| SD |  | 0.25 | 0.70 | 0.79 |
| Range |  | 0.38-1.15 | 1.59-2.99 | 0.38-2.99 |
| Maximum Expiratory Pressure (MEP, cm H_2_O) | | | | |
| N |  | 9 | 4 | 13 |
| Mean |  | 33.4 | 64.0 | 42.8 |
| SD |  | 10.0 | 15.9 | 18.6 |
| Range |  | 22-51 | 42-79 | 22-79 |
| Maximum Inspiratory Pressure (MIP, cm H_2_O) | | | | |
| N |  | 9 | 4 | 13 |
| Mean |  | -42.0 | -79.3 | -53.5 |
| SD |  | 17.8 | 24.9 | 26.3 |
| Range |  | -70-(-22) | -108-(-50) | -108-(-22) |

Table S2 shows baseline characteristics of the study population with regard to SMA type, maximum ulnar compound muscle action potential (CMAP) amplitude, modified Hammersmith Functional Motor Scale score (MHFMS); forced vital capacity (FVC); forced expiratory volume in one second (FEV1); maximum expiratory pressure (MEP); and maximum inspiratory pressure (MIP).
