# Supplementary material for: Phase II Open Label Study of Valproic Acid in Spinal Muscular Atrophy
Source: PLoS One. 2009 May 14;4(5):e5268. doi: 10.1371/journal.pone.0005268 (PMC2680034; doi:10.1371/journal.pone.0005268)
Supplement: Table S3 — (0.04 MB DOC) [file pone.0005268.s007.doc]

| Table S3: Relationship between SMN levels and SMN2 Dosage at Baseline | | |
| --- | --- | --- |
| SMN2 Copies | 3 | 4 |
| Full-length SMN referenced to pgk (flSMN/pgk) | | |
| N | 25 | 7 |
| Mean | 0.69 | 0.82 |
| SD | 0.38 | 0.78 |
| Range | 0.13-1.50 | 0.36-2.55 |
| Delta 7 SMN (Δ7SMN) referenced to pgk (Δ7SMN/pgk) | | |
| N | 25 | 7 |
| Mean | 1.10 | 1.16 |
| SD | 0.60 | 1.05 |
| Range | 0.37-2.71 | 0.40-3.38 |
| Full-length SMN (flSMN) referenced to po (flSMN/po) | | |
| N | 28 | 9 |
| Mean | 0.67 | 0.79 |
| SD | 0.29 | 0.08 |
| Range | 0.05-1.27 | 0.66-0.92 |
| Delta 7 SMN (Δ7SMN) referenced to po (Δ7SMN/po) | | |
| N | 28 | 9 |
| Mean | 1.04 | 1.15 |
| SD | 0.53 | 1.24 |
| Range | 0.36-2.14 | 0.05-4.29 |

po = human RPLPO (large ribosomal protein) and pgk1 = human phosphoglycerate kinase 1, which served as endogenous controls for determining relative amounts of SMN transcripts.

Table S3: Relationship between SMN levels and SMN2 Dosage at Baseline. Data provided is referenced to two different endogenous controls: human RPLPO and pgk1.
